# Optimization of Liquid Fermentation of *Acanthopanax senticosus* Leaves and Its Non-Targeted Metabolomics Analysis

**DOI:** 10.3390/molecules29194749

**Published:** 2024-10-08

**Authors:** Rui Zhang, Xueyan Wang, Jiaojiao Xue, Xiaoli Li, Ying Li, Yi Ding, Yichao Feng, Xueping Zhang, Jianqing Su, Xiuling Chu

**Affiliations:** College of Agronomy and Agricultural Engineering, Liaocheng University, Liaocheng 252000, China; 17863709708@163.com (R.Z.); wangxueyan202203@163.com (X.W.); x15065441211@163.com (J.X.); lxl15006995983@163.com (X.L.); ly15963373832@163.com (Y.L.); djy15373023047@163.com (Y.D.); 15553170728@163.com (Y.F.); 13562285648@163.com (X.Z.)

**Keywords:** *Acanthopanax senticosus* leaves, fermentation, complex probiotic, non-targeted metabolomics

## Abstract

To enhance the nutritional value of *Acanthopanax senticosus* leaves (AL), a fermentation process was conducted using a probiotic *Bacillus* mixture, and the changes in chemical constituents and biological activities before and after fermentation were compared. A response surface methodology was employed to optimize the liquid fermentation conditions of AL based on their influence on polyphenol content. Non-targeted metabolomics analysis was performed using LC-MS/MS to reveal the differing profiles of compounds before and after fermentation. The results indicated that *Bacillus subtilis* LK and *Bacillus amyloliquefaciens* M2 significantly influenced polyphenol content during fermentation. The optimal fermentation conditions were determined to be a fermentation time of 54 h, a temperature of 39.6 °C, and an inoculum size of 2.5% (*v*/*v*). In comparison to unfermented AL, the total polyphenol and flavonoid contents, as well as the free radical scavenging capacities measured by DPPH and ABTS assays, and the activities of β-glucosidase and endo-glucanase, were significantly increased. The non-targeted metabolomics analysis identified 1348 metabolites, of which 829 were classified as differential metabolites. A correlation analysis between the differential metabolites of polyphenols, flavonoids, and antioxidant activity revealed that 13 differential metabolites were positively correlated with antioxidant activity. Kyoto encyclopedia of genes and genomes (KEGG) enrichment analysis of the differential metabolites identified 82 pathways, with two of the top 25 metabolic pathways related to flavonoids. This study explores the potential for enhancing the active ingredients and biological effects of AL through probiotic fermentation using *Bacillus* strains.

## 1. Introduction

*Acanthopanax senticosus* leaves (AL), recognized for their dual role in medicine and nutrition, possess a wide range of biological properties [1]. Research has demonstrated that AL contain various active constituents, including polyphenols, polysaccharides, and flavonoids [2,3,4]. These compounds have been shown to be effective in the treatment of cardiovascular diseases and hypoglycemia [5], as well as exhibiting anti-inflammatory [6], antioxidant [7], and immunomodulatory [8] effects. Shi et al. [9] found that the extract of AL enhanced the activities of superoxide dismutase (SOD) and catalase (CAT) in serum, indicating a strong in vivo antioxidant capacity. Additionally, Hu et al. [10] analyzed the phenolic constituents of AL and identified 20 phenolic compounds, which were confirmed to be associated with hypoglycemic activity through in vitro assays.

Microbial fermentation is a widely used food processing technology [11]. During liquid fermentation, hydrolytic enzymes produced by microbial metabolism break down plant cell walls, facilitating the release of active ingredients into the fermentation broth. In contrast, microbial metabolites are dissolved within this broth. Fermentation not only liberates the active components of the substrate but also enhances its original properties or generates new effects [12]. Karina et al. [13] indicated that the content of polyphenols, flavonoids, and other active substances in the substrate was enhanced by fermentation with bacteria such as *Bacillus*. Rhujuk/Bastanga was found to have significantly higher levels of phenolic content (1.44 mg GAE/g and 2.44 mg GAE/g), thus having high antioxidant activity in comparison to the other fermented products [14]. *Bacillus* has a short growth cycle, is easy to cultivate, demonstrates greater adaptability to external environments, and can survive in relatively unfavorable temperature and pH rang [15]. However, to date, no studies have investigated the impact of *Bacillus* strains fermentation on the quality of AL.

Currently, non-targeted metabolomics is employed to study metabolites, using common analytical methods including gas chromatography-tandem mass spectrometry (GC-MS/MS), liquid chromatography-tandem mass spectrometry (LC-MS/MS), and nuclear magnetic resonance (NMR) [16]. Among these methods, LC-MS/MS is particularly favored due to its high resolution, sensitivity, and broad applicability [17], making it suitable for exploratory studies of unknown metabolites in specimens using non-targeted techniques. This method has been extensively utilized to analyze the chemical composition and metabolite content of various substances [18]. Mok et al. [19] inoculated *Bacillus subtilis* WX-17 into soybean dregs for liquid fermentation. They conducted a non-targeted metabolomics analysis and discovered that liquid fermentation significantly increased the levels of amino acids, short-chain fatty acids, and other valuable metabolites. Additionally, non-targeted metabolomics analysis revealed significant changes in secondary metabolites from the third to the fifth day after instant green tea was liquid fermented by *Eurotium cristatum* [20]. Zhao et al. [21] analyzed extracts from the liquid fermentation of barley by *Lactobacillus plantarum* dy-1 using non-targeted metabolomics, detecting a total of 124 metabolites, which primarily included esters, amino acids, organic acids, and other bioactive compounds.

In this study, the liquid fermentation of AL with two *Bacillus* strains was investigated. Response surface methodology was employed to optimize the fermentation process based on polyphenol yields. During fermentation, the flavonoid and polyphenol contents, as well as the activities of β-glucosidase and endo-glucanase, and the free radical scavenging capacity of fermented *Acanthopanax senticosus* leaves (FAL), were compared with AL. Non-targeted metabolomics was utilized to analyze changes in differential metabolites before and after fermentation. This research aims to provide new insights into the biotransformation of the active constituents of AL and to establish a scientific foundation for the further development and utilization of AL.

## 2. Results and Discussion

### 2.1. Results of Screening for Dominant Strains

**Figure 1:**
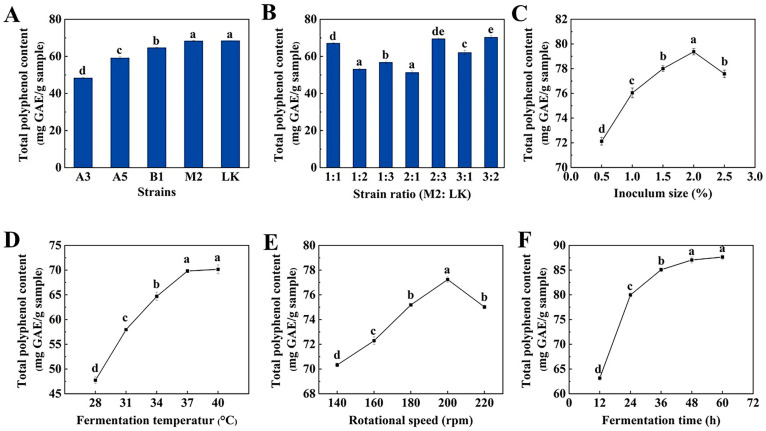
(**A**) Effect of single-strain fermentation on TPC. (**B**) Effect of fermentation with different strain ratios on TPC. (**C**) Effect of inoculum size on TPC. (**D**) Effect of fermentation temperature on TPC. (**E**) Effect of rotational speed on TPC. (**F**) Effect of fermentation time on TPC. Values are expressed as mean ± standard deviation (*n* = 3). Note: a, b, c, d, and e represent the significance of the difference within the graph. Containing the same letter means the difference is insignificant (*p* > 0.05).

Different strains of bacteria exhibit varying abilities to utilize nutrients in the fermentation medium, making it essential to screen for strains suitable for the fermentation of AL. As illustrated in Figure 1A, among the five *Bacillus* species examined, *Bacillus subtilis* LK and *Bacillus amyloliquefaciens* M2 produced the highest total polyphenol content (TPC) of 68.40 mg GAE/g sample and 68.30 mg GAE/g sample, respectively. Consequently, *Bacillus subtilis* LK and *Bacillus amyloliquefaciens* M2 were selected as the dominant strains for subsequent studies.

### 2.2. Optimization of Strains Ratio

Mixture strain fermentation systems are generally superior to single-strain systems due to the reciprocal symbiotic relationships among the strains [22]. As shown in Figure 1B, when the ratio of *Bacillus amyloliquefaciens* M2 to *Bacillus subtilis* LK was 3:2, the highest TPC in FAL was 70.32 mg GAE/g of sample, significantly surpassing the TPC observed in fermentations using M2 or LK alone. Fermentation processes are driven by either simple or complex microbial communities that interact with one another, while also engaging with the substrate. The mechanisms underlying microorganism-to-microorganism interactions are intricate, and composite inoculated fermentations typically outperform single-organism fermentations [23]. As reported by Lin et al. [24], the flavonoid content following mixed fermentation with yeast, *Bacillus subtilis*, and *Lactobacillus* (61.49 ± 0.53 mg/mL) was significantly higher than the flavonoid content resulting from fermentations with yeast (51.14 ± 0.39 mg/mL), *Bacillus subtilis* (55.82 ± 0.77 mg/mL), or *Lactobacillus* (44.76 ± 0.78 mg/mL) when fermented individually, indicating that the three probiotics functioned as mutual promoters.

### 2.3. Single-Factor Test

Figure 1C demonstrates the effect of inoculum amount on FAL. TPC was highest at an inoculum size of 2.0% (*v*/*v*). At low inoculum size, probiotics secrete less initial cellulase, leading to slower cellulose degradation. Conversely, at high inoculum size, probiotics rapidly consume nutrients during the early stages of fermentation [25], indicating that inoculum size significantly influences the growth and metabolism of probiotics. At fermentation temperatures (Figure 1D), TPC during fermentation increased from 28 °C to 37 °C, after which the curves flattened. At lower temperatures, the mobility of microbial cell membranes decreases, inhibiting their growth [26]. Conversely, excessively high temperatures can lead to premature senescence and death of microorganisms. Therefore, temperature is a critical factor in the fermentation process. The rotational speed also significantly influenced the TPC in the FAL (Figure 1E). It increased markedly when the rotational speed ranged from 140 rpm to 200 rpm, but subsequently decreased as the rotational speed continued to rise. The rotational speed correlates with the fermentation system’s dissolved oxygen status, and strains can grow better at appropriate dissolved oxygen concentrations [27]. At low rotational speeds, the concentration of dissolved oxygen in the fermentation system becomes insufficient, adversely affecting the strain’s metabolism. Conversely, excessively high rotational speeds enhance the oxygen dissolution rate, which can lead to the strain rapidly entering the attenuation stage [28]. Fermentation is a process that involves the growth and metabolism of microorganisms, as well as the degradation of various substances, necessitating a specific duration. However, extending the fermentation time may lead to increased costs and other complications, therefore, selecting the appropriate fermentation duration is particularly crucial [25]. The impact of fermentation time on TPC in FAL is illustrated in Figure 1F. There was a significant increase in TPC in the range of 12 h–48 h; in the 48 h to 60 h range, there was a tendency for TPC to increase, but this was not statistically significant. In conclusion, considering economic factors, the optimal fermentation conditions identified were an inoculum size of 2.0% (*v*/*v*), a fermentation temperature of 37 °C, a rotational speed of 200 rpm, and a fermentation time of 48 h.

### 2.4. Response Surface Methodology

From the results of the single-factor test, it is evident that fermentation time, fermentation temperature, and inoculum amount have a more significant effect on TPC. Consequently, these three factors were chosen as independent variables for the box-behnken design assay. The experimental design and results are shown in Table 1. As shown in Table 2, the model for TPC in the ANOVA was highly significant (*p* < 0.0001), indicating that TPC varied significantly across different conditions. The lack of fit is insignificant (0.0899 > 0.05), meaning the regression model is reasonable. The R^2^ value of 0.9852 indicates that the model is well-fitted. The difference between Adj R^2^ (0.9661) and Pred R^2^ (0.8114) is less than 0.2, indicating that the model is feasible. Adeq precision (23.6824 > 4.0) showed that the regression model had an excellent predictive effect. The effects of B, AB, AC, A^2^, and B^2^ on TPC in the model were highly significant (*p* < 0.01); C was significant (*p* < 0.05).

**Figure 2:**
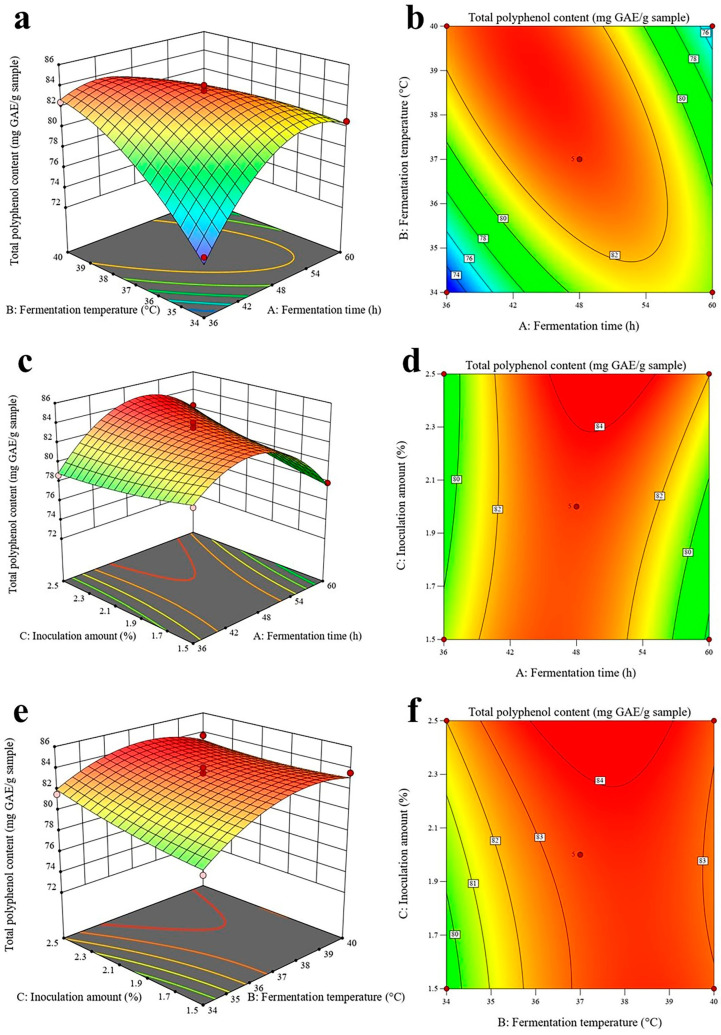
Response surface plots. (**a**,**b**) show the interaction between fermentation time and fermentation temperature; (**c**,**d**) show the interaction between fermentation time and inoculation amount; (**e**,**f**) show the interaction between fermentation temperature and inoculation amount. Note: red dot represents above surface; pink dot represents below surface.

Based on the obtained quadratic multiple regression equations, 3D response surface plots of the three influencing factors of TPC were produced using Design-Expert 13 software, and the results are shown in Figure 2. This figure reflects the dynamics of the two-by-two interaction among the three influencing factors on TPC, and the optimal values of these factors during the incubation of FAL. A steeper 3D response surface plot indicates a greater influence of a particular factor on the response surface value. The contour lines in the plot represent the interaction between the two variables; the closer the contour lines are to each other, the stronger the interaction between the two factors [29]. As shown in Figure 2a,b, the response surface plots were steeper, and the contour lines were denser, indicating that the interaction between fermentation time and fermentation temperature significantly affected TPC. The results of the response surface plot analysis were consistent with the ANOVA findings. In Figure 2, the model predicting the FAL polyphenol-producing capacity identified the optimal fermentation parameters: a fermentation time of 53.88 h, a fermentation temperature of 39.64 °C, and an inoculum size of 2.5%. Under these conditions, the expected polyphenol yield was 81.67 mg GAE/g of sample. Based on this optimal process prediction, and considering practical operability, the influencing factors were carefully controlled, and the optimal culture conditions were adjusted to a fermentation time of 54 h, a fermentation temperature of 39.6 °C, and an inoculum size of 2.5%. Three validation experiments were conducted to assess the accuracy of the predicted process. Referring to the predicted process, FAL yielded TPC of 82.69 mg GAE/g of sample, which represented an increase of 41.19 mg GAE/g of sample compared to the TPC yielded by AL. The test results closely aligned with the predicted outcomes. Therefore, the response surface methodology employed in this study to enhance TPC is both feasible and accurate.

### 2.5. Dynamic Changes of TPC and Total Flavonoid Content (TFC) at Different Fermentation Times

**Figure 3:**
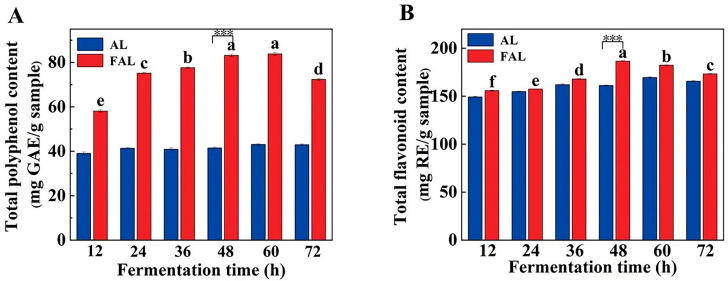
Effect of fermentation time on total polyphenol content (**A**) and total flavonoid content (**B**). Values are expressed as mean ± standard deviation (*n* = 3). Note: a, b, c, d, e, and f represent the significance of the difference within the graph. Containing the same letter means the difference is insignificant (*p* > 0.05). ***: *p* < 0.001.

Plants contain a diverse array of phenolic compounds, including flavonoids [30]. As in most plant stems and leaves, polyphenols and flavonoids in AL existing in bound and free forms [31]. During fermentation, probiotics produce metabolites that hydrolyze the molecules present in bound form, converting them into a free state and thereby enhancing the utilization of phenolic compounds [32]. Figure 3 shows that the overall trend of TPC and TFC for FAL was consistent with an initial increase followed by a decrease. Notably, we observed a significant increase in TPC and TFC in FAL compared to AL (*p* < 0.05). At 48 h, the polyphenol content of FAL was twice as high as that of AL, and the flavonoid content was 1.16 times greater than that of AL. Although TPC and TFC eventually declined, there was a significant improvement during the short-term fermentation period (0–48 h), which aligns with the findings of Wang et al. [33]. The increase in TPC and TFC during this initial phase may be attributed to the rapid growth of microorganisms that degrade cellulose, protein, pectin, and other components of FAL, subsequently leading to the release of polyphenols and flavonoids [34]. As fermentation progresses, the substances released are utilized, degraded, and transformed by microorganisms, resulting in a decrease in TPC and TFC [35].

### 2.6. Enzyme Activity Assay

**Figure 4:**
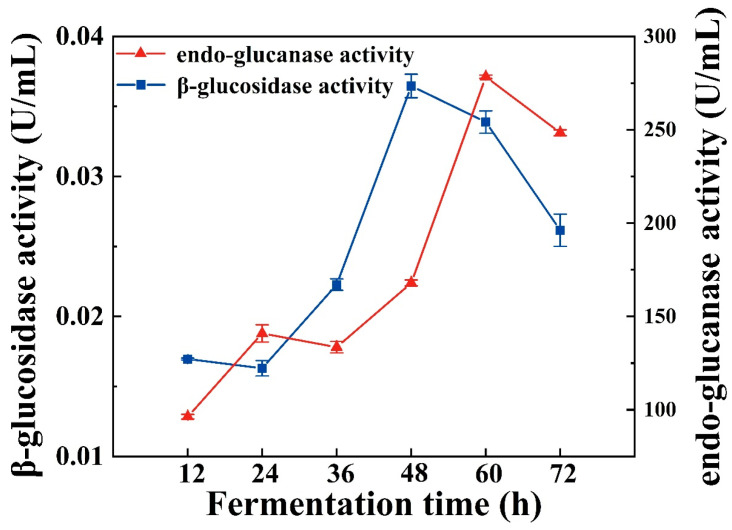
Effect of fermentation time on β-glucosidase activity and endo-glucanase activity. Values are expressed as mean ± standard deviation (*n* = 3).

*Bacillus* strains fermentation has been reported to produce a variety of hydrolytic enzymes, such as endo-glucanase [36], β-glucosidases [37], and proteases [38]. These enzymes are associated with the release of active substances from plants [39]. Among them, β-glucosidase is a crucial enzyme in carbohydrate metabolism, as it can cleave β-glucosidic bonds, thereby disconnecting oligosaccharides and other glucose conjugates. β-Glucosidase plays a vital role in numerous biological processes [40], including cellulose degradation [41]. The content of polyphenols and flavonoids in this study gradually increased over the 48 h of fermentation, peaking at 48 h (Figure 3A,B). Meanwhile, β-glucosidase activity remained low during the first 24 h of fermentation but then increased rapidly, reaching a maximum value of 0.03646 U/mL at 48 h (Figure 4). Additionally, endo-glucanase activity began to rise at 36 h, ultimately reaching a maximum value of 278.34 U/mL at 60 h (Figure 4). Endo-glucanase can disrupt the bond between phenolics and structural carbohydrates, thereby facilitating the release of insoluble phenolics [42]. Notably, the variations in β-glucosidase and endo-glucanase activities aligned with the trends observed in TPC and TFC, suggesting that both β-glucosidase and endo-glucanase—particularly the latter—play a crucial role in the AL fermentation process. These findings indicate that enhancing the activity of hydrolytic enzymes during fermentation may be a vital strategy for releasing phenolic compounds from AL.

### 2.7. Scanning Electron Microscopy (SEM)

**Figure 5:**
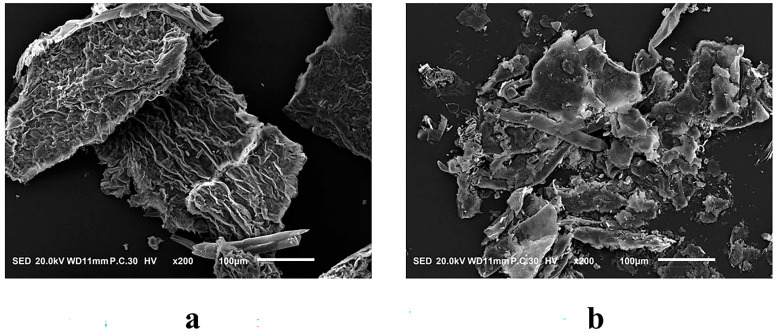
SEM of AL before and after fermentation. (**a**) unfermented; (**b**) fermented using *Bacillus subtilis* LK and *Bacillus amyloliquefaciens* M2.

To investigate the potential of hydrolytic enzymes produced by the probiotic mixture to catalyze the release of phenolics from AL, we examined the surface morphology of AL before and after fermentation using SEM (Figure 5). The electron micrographs of AL revealed that its cellulose walls were neatly arranged and dense, exhibiting a massive structure. The surfaces appeared almost intact (Figure 5a). In contrast, the microstructure of FAL underwent significant changes, as illustrated in Figure 5b. The microscopic surface of FAL was irregular and fragmented into small pieces, with the dense cellulose wall compromised by hydrolysis. Previous studies have indicated that phenolics primarily bind covalently to structures within plant cell walls [43]. Based on the data of TPC, TFC, and two hydrolytic enzymes in Section 2.5 and Section 2.6, it can be inferred that the enzymes produced by *Bacillus* strains disrupt the cellular structure of AL and hydrolyze the covalent bonds between polyphenolic compounds and the cell wall, ultimately facilitating the release of phenolic compounds [44].

### 2.8. Antioxidant Activity Analysis

**Figure 6:**
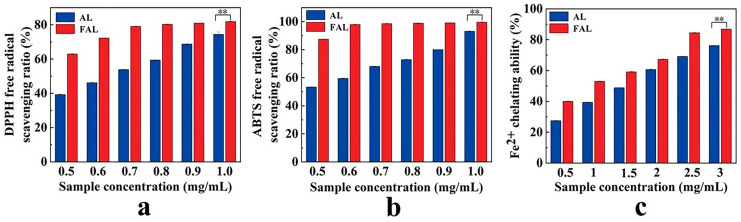
Effect of fermentation on the antioxidant activity of AL. (**a**) DPPH free radical scavenging ratio, (**b**) ABTS free radical scavenging ratio, (**c**) Fe^2+^ chelating activity. Values are expressed as mean ± standard deviation (*n* = 6). **: *p* < 0.01.

To investigate the effects of fermentation on the antioxidant activity of AL, we assessed the free radical scavenging capacity using the DPPH and ABTS assays, and measured the Fe^2+^ chelating activity through a colorimetric method. The DPPH and ABTS assays evaluate antioxidant activity based on the reaction of antioxidants with organic free radicals [45]. Additionally, the presence of other chelating agents that interfere with the Fe^2+^-ferrozine complex was identified through the measurement of Fe^2+^ chelating activity [46]. Figure 6a shows the scavenging of DPPH radicals by FAL and AL increased from 63.11% and 39.31% to 81.94% and 72.32%, respectively, when the sample concentration was raised from 0.5 mg/mL to 1.0 mg/mL. The IC_50_ values were calculated, revealing that the IC_50_ for FAL was 0.320 mg/mL, while for AL it was 0.636 mg/mL, which was 1.98 times higher than that of FAL. This indicates that FAL possesses a stronger scavenging ability for DPPH radicals compared to AL. As depicted in Figure 6b, both AL and FAL demonstrated concentration-dependent scavenging, with an increase in the scavenging of ABTS radicals corresponding to higher sample concentrations. The scavenging of ABTS radicals by FAL and AL rose from 87.64% and 53.78% to 99.60% and 92.99%, respectively, as the sample concentration increased from 0.5 mg/mL to 1.0 mg/mL. The calculated IC_50_ values for ABTS were 0.304 mg/mL for FAL and 0.507 mg/mL for AL, which was 1.67 times higher than that of FAL. This result further indicated that FAL exhibits superior scavenging ability for ABTS radicals compared to AL. Figure 6c shows the Fe^2+^ chelating activity of FAL and AL, which increased from 40.03% and 27.45% to 86.84% and 76.20%, respectively, as the sample concentration was gradually increased from 0.5 to 3.0 mg/mL. The IC_50_ values for Fe^2+^ chelating activity was found to be 0.841 mg/mL for FAL and 1.325 mg/mL for AL, which was 1.58 times higher than that of FAL. Previous research has established that polyphenols possess antioxidant properties [47], and there is a significant positive linear correlation between TPC and antioxidant activity.

### 2.9. Non-Targeted Metabolomics

#### 2.9.1. Effect of Fermentation with Mixture Probiotic on AL Metabolites

**Figure 7:**
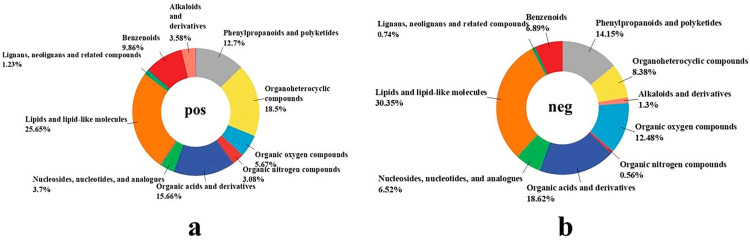
Schematic representation of the different classifications of FAL metabolites. (**a**) Metabolites in positive ion mode; (**b**) metabolites in negative ion mode.

To further elucidate the metabolite changes during AL fermentation, we employed an LC-MS/MS-based approach to investigate the metabolites. The metabolite structures of the samples were characterized by matching the basic information of the metabolites in the database, including mass-to-charge ratio (m/z), retention time (RT), and molecular weight. In present study, we identified a total of 1348 metabolites, including 811 in the positive ion mode and 537 in the negative ion mode. In order to visualize the composition and classification of these metabolites, the corresponding pie charts are presented in Figure 7. Different colors in each pie chart represent various classifications, while the regions indicate the relative proportions of metabolites within each classification. Figure 7 shows the major superclasses of metabolites, including lipids and lipid-like molecules, organoheterocyclic compounds, phenylpropanoids and polyketides, organic acids and derivatives, and benzenoids.

**Figure 8:**
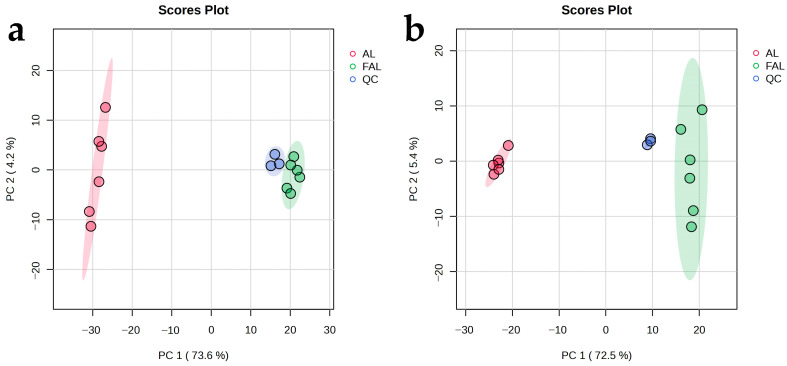

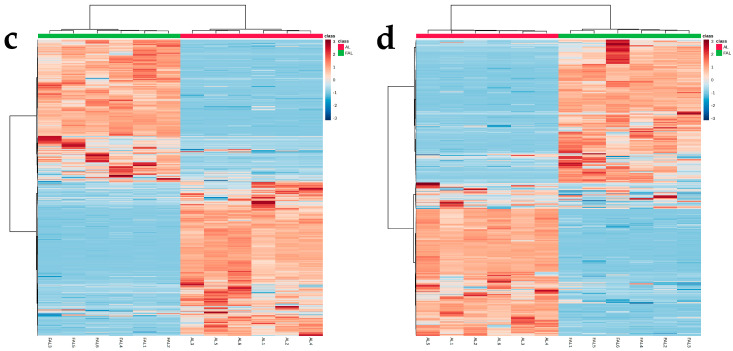
PCA and hierarchical clustering heatmap showing changes in metabolites during AL fermentation. (**a**) PCA score plot in positive ion mode; (**b**) PCA score plot in negative ion mode; (**c**) hierarchical clustering heat map in positive ion mode; (**d**) hierarchical clustering heat map in negative ion mode.

In order to scientifically identify the differences in metabolite accumulation before and after the fermentation of AL, we conducted an analysis of the metabolic constituents using principal component analysis (PCA) [48]. Each scatter point represents an individual sample, with the color of the point indicating different groupings. A closer distribution of sample points suggests greater similarity in the type and content of metabolites between samples; conversely, a wider distribution indicates a more significant difference in their overall metabolic levels. In Figure 8a,b, the quality control (QC) samples in both positive and negative ion modes were tightly clustered and separated from the AL and FAL samples, suggesting that the LC-MS/MS method has excellent overlap, stability, and significant changes in AL and FAL metabolites. To further analyze the changes in metabolites before and after the fermentation of AL, a hierarchical cluster analysis was conducted. The results are represented in color-coded classes, ranging from red to blue, which indicate the relative abundance of metabolites from high to low. As shown in Figure 8c,d, the metabolites were changed by the fermentation of the complex bacteria.

#### 2.9.2. Differential Metabolite Analysis Based on Supervised Orthogonal Partial Least Squares Discriminant Analysis (OPLS-DA)

**Figure 9:**
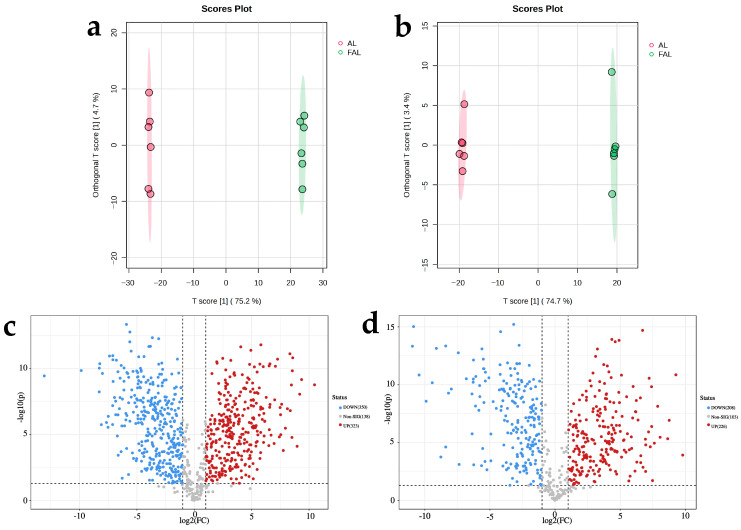
OPLS-DA score plots and volcano plots showing changes in metabolites during AL fermentation. (**a**) OPLS-DA scores plot in positive ion mode; (**b**) OPLS-DA scores plot in negative ion mode; (**c**) volcano plot in positive ion mode; (**d**) volcano plot in negative ion mode.

Typically, metabolomics data are characterized by small samples (small molecular weights detected) and high dimensionality (large variety of metabolites identified) [49]. Consequently, among these variables, there are both discrepant variables related to categorical variables and a significant number of undifferentiated variables that may be correlated with one another. This correlation can lead to the dispersion of discrepant variables across multiple PC when analyzed using the PCA model, due to the identification of fundamental differences among sample groups, and it complicates the screening of effective discrepant metabolites. In contrast, OPLS-DA effectively eliminates information from metabolites that are irrelevant to categorical variables, thereby facilitating a more accurate analysis of intergroup differences within the model [50]. The horizontal co-ordinate T score [1] of the OPLS-DA score plot in Figure 9 represents the value of the main component score in quadrant 1, demonstrating between-sample group differences, and the vertical co-ordinate Orthogonal T score [1] describes the value of the orthogonal PC score in quadrant 1, explaining within-sample group differences. As shown in Figure 9a,b, the OPLS-DA score plots in both positive and negative ion modes exhibited significant differences between groups, with all samples falling within the 95% confidence interval. This indicates notable differences in the metabolites produced during AL fermentation, which can be utilized in the subsequent analysis of the differential components. To illustrate the variability of metabolites, volcano plots were generated using statistical values of *p* < 0.05 and fold change (FC) ≥ 2 or ≤0.5 (Figure 9c,d). The results indicated that 323 metabolites were significantly upregulated and 350 metabolites were significantly downregulated in positive ion mode, while 226 metabolites were significantly upregulated and 208 metabolites were significantly downregulated in negative ion mode.

#### 2.9.3. Correlation of Polyphenols and Flavonoids Differential Metabolites with Antioxidant Activity of AL and FAL

**Figure 10:**
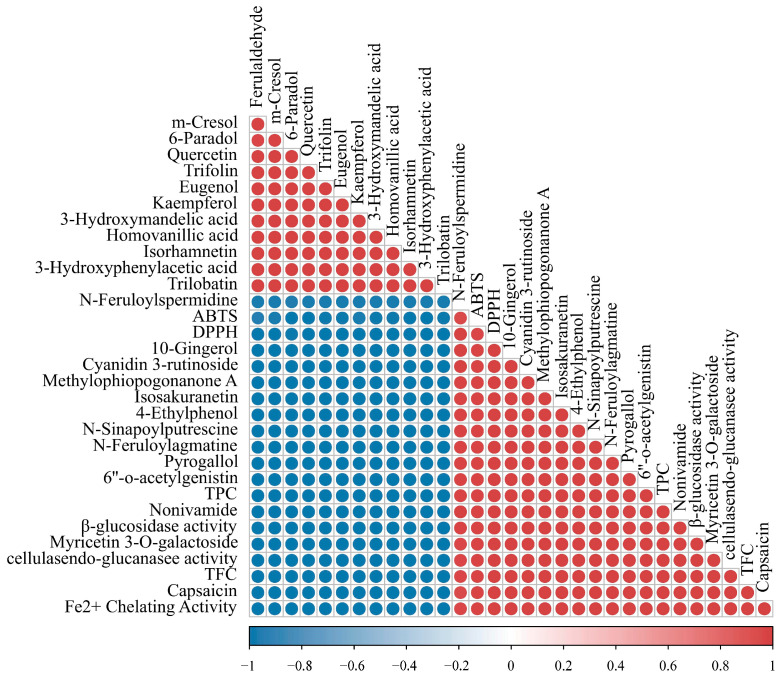
Correlation plot between differential metabolites in polyphenols and flavonoids, and antioxidant activity (DPPH, ABTS, and Fe^2+^ chelating activity) and enzyme activity (endo-glucanase, β-glucosidase), with the colour of the circles indicating the correlation coefficients and the magnitude of the values within the circles indicating the statistical differences, with the red circles indicating a positive correlation, and the blue circles indicating a negative correlation.

**Figure 11:**
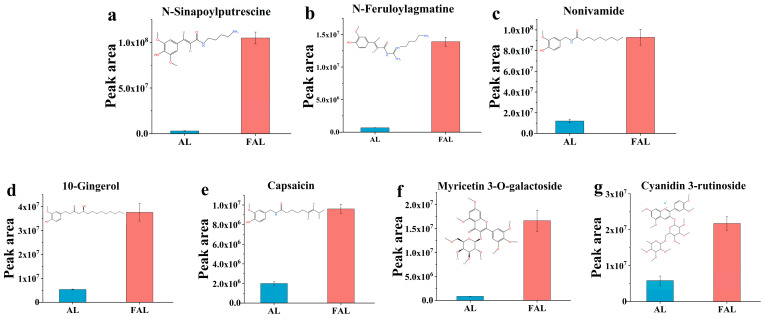
(**a**–**e**) Histograms of possible structures and relative peak intensities of five methoxyphenols; (**f**,**g**) histograms of possible structures and relative peak intensities of two flavonoid glycosides.

The differential metabolites were screened based on variable importance in projection (VIP) > 1, *t*-test results *p* < 0.001, and FC ≥ 2 or ≤ 0.5 among the total metabolites. This analysis identified 829 differential metabolites, which included 15 polyphenols and 45 flavonoids. To investigate the differential polyphenolic and flavonoid metabolites of AL and FAL, and their relationship to antioxidant activity, the differential flavonoid metabolites were ranked according to log2(FC), and the top 10 metabolites with the highest multiplicity differences (both upregulated and downregulated) were selected. The differential polyphenol metabolites, differential flavonoid metabolites, and indices of antioxidant activity were subjected to Pearson’s correlation coefficient (r) analysis. The results are shown in Figure 10. The figure illustrates that 13 metabolites exhibited a positive correlation with antioxidant activity, while 12 metabolites demonstrated a negative correlation. Furthermore, TPC and TFC were positively correlated with the antioxidant indices, thereby confirming that an increase in polyphenol and flavonoid content enhances antioxidant capacity. The 13 metabolites positively correlated with the antioxidant indices included six methoxyphenols, two flavonoid glycosides, one 1-hydroxy-2-unsubstituted benzenoid, one benzenetriol and derivative, one isoflavonoid o-glycoside, one homoisoflavan, and one flavone. Previous studies have indicated that methoxyphenols and their derivatives possess antioxidant activity [51]. Figure 11a–e demonstrate that fermentation significantly increases the content of five types of methoxyphenol: n-sinapoylputrescine by 35.27-fold, n-feruloylagmatine by 20.75-fold, nonivamide by 7.72-fold, 10-gingerol by 6.99-fold, and capsaicin by 4.81-fold. Research indicates that the antioxidant activity of flavonoid glycosides is influenced by the number of hydroxyl substituents in their structure; generally, a higher number of hydroxyl groups correlates with stronger antioxidant activity [52]. As illustrated in Figure 11f,g, fermented AL enhances the flavonoid glycoside content in the fermentation broth, resulting in a 19.70-fold increase in myricetin 3-o-galactoside and a 3.72-fold increase in cyanidin 3-rutinoside. The correlation with antioxidant activity follows the order: cyanidin 3-rutinoside > myricetin 3-o-galactoside (Figure 10). Notably, a greater number of hydroxyl substituents corresponds to higher r values, as suggested by the structural diagrams in Figure 11f,g.

#### 2.9.4. Correlation of Polyphenol and Flavonoid Differential Metabolites with Enzyme Activities

To investigate the relationship between polyphenol and flavonoid differential metabolites and enzyme activities, Pearson’s correlation coefficients were analyzed between endo-glucanase activity, β-glucosidase activity, and the 25 differential metabolites identified in Section 2.9.3. The results are presented in Figure 10. The result showed a positive correlation between endo-glucanase activity, β-glucosidase activity, and TPC and TFC. This suggests that the enzymes produced by probiotics during fermentation promote the release of active compounds from the AL [53]. It was observed that, in the presence of β-glucosidase, soy isoflavone glucosides are converted into glycosides [54]. As shown in Figure 10, the content of trilobatin and trifolin decreased after fermentation, which was negatively correlated with β-glucosidase activity, indicating that β-glucosidase activity may convert trilobatin and trifolin. However, no increase in the corresponding glycosides was detected, likely due to the minimal differences in glycoside concentrations when screening for differentiated flavonoid metabolites.

#### 2.9.5. KEGG Enrichment Analysis of Differential Metabolites

**Figure 12:**
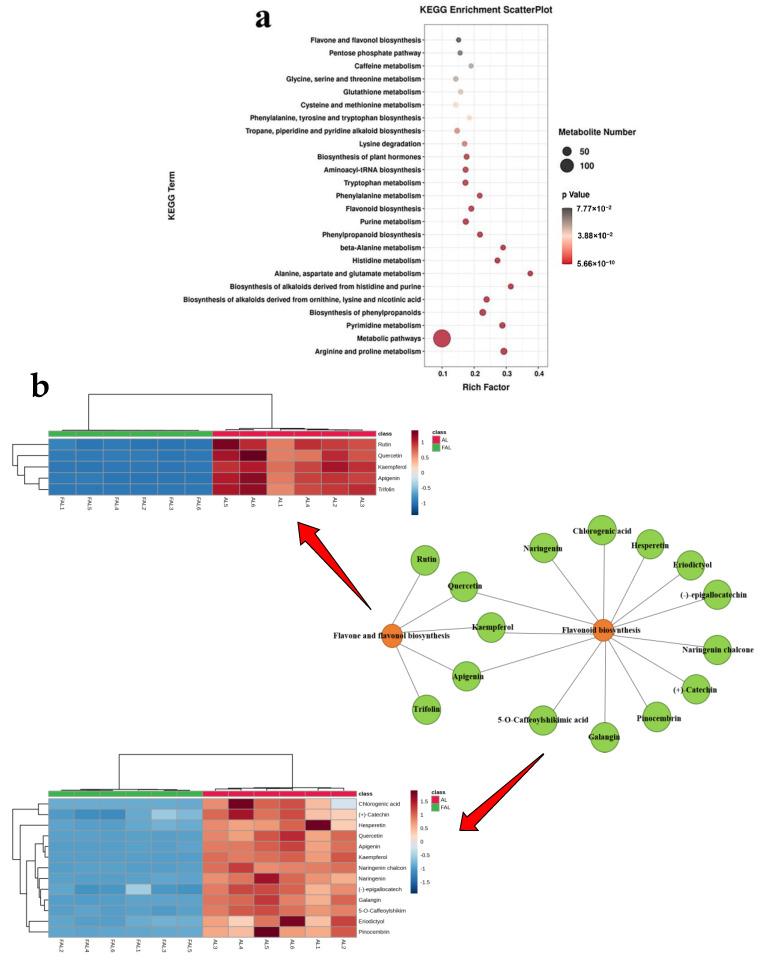
KEGG enrichment pathway based on differential metabolites between AL and FAL. (**a**) Pathway enrichment analysis. Each bubble in the bubble diagram represents a metabolic pathway, and bubble size is proportional to enrichment. The top 25 items with the highest *p*-values were selected; (**b**) network diagram of associations and metabolite heatmap. Orange nodes indicate metabolic pathways and green nodes indicate metabolites.

The differential metabolites were entered into the KEGG database for pathway enrichment analysis. The results identified 82 enriched pathways, with the top 25 selected based on their *p*-values. As shown in Figure 12a, the majority of the metabolites were enriched in “metabolism” and “biosynthesis” pathways, including arginine and proline metabolism, alanine, aspartate, and glutamate metabolism, biosynthesis of plant hormones, and flavonoid biosynthesis. This suggests that the mixture probiotic fermentation altered the metabolite composition of the AL. Flavonoids, as primary secondary metabolites of plants, play a crucial role in antioxidant activity [55,56]. Figure 12b illustrates a network diagram depicting the relationship between two metabolic pathways: flavonoid biosynthesis and the biosynthesis of flavonoids and flavonols, alongside the differential metabolites identified in the heat map. Among these metabolites, quercetin, apigenin, kaempferol, naringenin, chlorogenic acid, hesperetin, eriodictyol, naringenin chalcone, (+)-catechin, pinocembrin, galangin, 5-o-caffeoylshikimic acid, and (-)-epigallocatechin are associated with flavonoid biosynthesis. In contrast, quercetin, apigenin, kaempferol, rutin, and trifolin are linked to the biosynthetic pathways of flavonoids and flavonols. Overall, the results of KEGG enrichment analysis further revealed the changes brought about by the AL fermentation of the mixed *Bacillus* species, demonstrating the diversity of metabolic pathways during the fermentation process.

## 3. Materials and Methods

### 3.1. Materials and Chemicals

AL was purchased from Baishan Pharmaceutical Co. Ltd. (Baishan, Jilin Province, China). The fresh AL was dried in a constant-temperature blast dryer at 65 °C, pulverized using a pulverizer, sieved through a 60-mesh sieve, and stored at 4 °C for future use. Rutin, gallic acid, aluminum nitrate, sodium hydroxide, soybean meal, folin and ciocalteu’s phenol reagent, p-nitrophenyl-β-D-galactopyranoside (pNPG), sodium chloride, sodium nitrite, and sodium carbonate anhydrous were purchased from Shanghai Macklin Company Co., Ltd. Potassium persulfate and D-(+)-glucose were purchased from Aladdin Company Co., Ltd. (Shanghai, China). The Luria-Bertani (LB) medium was purchased from Qingdao HaiBo BioTechnology Co., Ltd. (Qingdao, China).

### 3.2. Fermentation Media and Strains

Fermentation medium: AL powder 1 g, soybean meal 1 g, D-(+)-glucose 1 g, sodium chloride 0.5 g, distilled water 100 mL.

*Cytobacillus oceanisediminis* A3, *Bacillus cereus* A5, *Bacillus subtilis* B1, *Bacillus subtilis* LK, and *Bacillus amyloliquefaciens* M2 were isolated by our laboratory.

### 3.3. Screening of Probiotics

Each of the five *Bacillus* species was inoculated into LB medium and activated at 37 °C for 24 h. Following activation, the cultures were centrifuged, washed twice, and resuspended in 50 mM sterile potassium phosphate buffer to achieve a concentration of approximately 1 × 10^7^ CFU/mL. The fermentation medium was sterilized by heating at 121 °C for 20 min, then cooled to room temperature. The bacterial solution was inoculated with the substrate at a 2% (*v*/*v*) inoculum and incubated in an oscillating incubator (Zhichu Instrumentation Co., Ltd., Shanghai, China) at 37 °C for 24 h. A fermentation medium without the inoculation of the strains served as a control. *Bacillus* strains were screened based on their ability to increase the TPC of the substrate, identifying those suitable for the fermentation of AL.

### 3.4. Optimization of Strain Ratio

Based on the experimental results from Section 2.3, M2: LK was optimized according to inoculation ratios of 1:1, 1:2, 1:3, 2:1, 2:3, 3:1, and 3:2. The effects of these different strain ratios on the production of TPC during fermentation were investigated.

### 3.5. Single-Factor Experiment

A single-factor test was conducted with four independent variables: inoculum size (1.0, 1.5, 2.0, 2.5, and 3.0%), fermentation temperature (28, 31, 34, 37, and 40 °C), rotational speed (140, 160, 180, 200, and 220 rpm), and fermentation time (12, 24, 36, 48, and 60 h). The TPC in the fermentation products was used as the dependent variable. The effects of these four factors on TPC production by AL during liquid fermentation were investigated.

### 3.6. Response Surface Methodology Assay

Concerning the results of the single-factor test, three factors—fermentation time (A), fermentation temperature (B), and inoculum amount (C)—were used as independent variables and fermentation product TPC (Y) was used as the response value. The response surface optimization design was carried out for the AL process of mixture probiotic liquid fermentation using the box-behnken design method through the Design-Expert 13.0 software, as shown in Table 3.

### 3.7. Dynamic Observation during Fermentation

The effect of different fermentation times on AL was determined according to the optimal fermentation process. TPC and TFC were measured at 12, 24, 36, 48, 60, and 72 h, while the activities of endo-glucanase and β-glucosidase were evaluated at the same time intervals.

### 3.8. Determination of TPC and TFC

TPC was determined according to the method in [57]. Gallic acid was used as a standard, and data are provided in milligrams of gallic acid equivalent (mg GAE/g sample).

TFC was determined using the method [58]. Rutin was used as a standard, and data are provided in milligrams of rutin equivalent (mg RE/g sample).

### 3.9. Enzyme Activity Assay

#### 3.9.1. Endo-Glucanase Activity

Endo-glucanase activity was determined according to previous methods [59] with slight modification. Definition of enzyme activity unit: the amount of enzyme required to catalyze the production of 1 μg reducing sugar per minute from 1% CMC-Na at 60 °C and pH 5.0 was taken as one enzyme activity unit U (μg·min^−1^·mL^−1^)

(1) $$enzyme\;activity(U)=\frac{N1\times N2\times G\times 1000}{10\times 0.8}$$

In the formula, G: the mass of glucose found from the standard curve (mg); N1: dilution times of fermentation solution; N2: dilution times of reaction solution during colorimetry; 1000: conversion of milligrams and micrograms; 10: reaction time of enzyme hydrolysis (min); 0.8: volume of fermentation solution used in the determination of enzyme activity (mL).

#### 3.9.2. β-Glucosidase Activity

Determination of β-glucosidase activity by reference to the previous method [59]. Enzyme activity unit (U): under the condition of 50 °C and pH 7.0, the enzyme required to generate 1 μmol of p-nitrophenol per milliliter of enzyme solution per minute is one enzyme activity unit.

### 3.10. SEM

The specific procedure was referred to as the method of Cheng et al. [60] with some modifications. The fermented samples were centrifuged, the supernatant was discarded, and the precipitate was washed twice with distilled water and then dried in a blast drying oven (Yiheng Scientific Instrument Co., Ltd., Shanghai, China) at 65 °C for 12 h. The fixed conductive adhesive was added to the dry AL or FAL powder, and this was stuck on the sample stage for gold spraying treatment; the gold spraying time was 30 s, and the measurement voltage was 10 kV. The morphology and structure of the sample were observed, and magnification was 200×.

### 3.11. Antioxidant Activity

Some modifications were made to the method for determining DPPH and ABTS radical scavenging activities, following the protocols established by Andrade et al. [61] and Jeon et al. [62]. For the determination of DPPH, 0.5 mL of a gradient concentration of the sample solution (0.5, 0.6, 0.7, 0.8, 0.9, 1.0 mg/mL) was mixed with 1 mL of DPPH solution (0.2 mM), and the absorbance was measured at 517 nm after dark treatment for 30 min. To determine ABTS, the ABTS stock solution was prepared by mixing equal volumes of ABTS solution (7 mM) and potassium persulfate solution (2.5 mM), then left at room temperature for 16 h, protected from light. The stock solution was diluted with anhydrous ethanol to achieve an absorbance of (0.7 ± 0.02) at 734 nm before use. Subsequently, 1 mL of the sample solution at gradient concentrations (0.5, 0.6, 0.7, 0.8, 0.9, 1.0 mg/mL) was mixed with 4 mL of ABTS solution, and the absorbance was measured at 734 nm after dark treatment for 6 min.

(2) $$DPPH\;free\;radical\;scavenging\;ratio\left(\%\right)=\frac{A_{1}-\left(A_{2}-A_{3}\right)}{A_{1}}\times 100\%$$

where, A_1_ is the absorbance of the control group (anhydrous ethanol + DPPH), A_2_ is the absorbance of the sample group (sample + DPPH), and A_3_ is the absorbance of the background group (sample + anhydrous ethanol).

(3) $$ABTS\;free\;radical\;scavenging\;ratio\left(\%\right)=\frac{B_{1}-\left(B_{2}-B_{3}\right)}{B_{1}}\times 100\%$$

where, B_1_ is the absorbance of the control group (anhydrous ethanol + DPPH), B_2_ is the absorbance of the sample group (sample + DPPH), and B_3_ is the absorbance of the background group (sample + anhydrous ethanol). 

The Fe^2+^ chelation activity was determined using the method described by Wang et al. [63]. In brief, 400 μL of a sample solution with varying concentrations (0.5, 1.0, 1.5, 2.0, 2.5, and 3.0 mg/mL) was combined with 50 μL of FeCl_2_ solution (2 mM), 100 μL of ferrozine solution (5 mM), and 2 mL of methanol. The absorbance was measured at 562 nm after allowing the reaction to proceed for 10 min at room temperature.

(4) $${Fe}^{2+}chelating\;activity\left(\%\right)=\frac{C_{1}-\left(C_{2}-C_{3}\right)}{C_{1}}\times 100\%$$

where, C_1_ is the absorbance of the control group, C_2_ is the absorbance of the sample group, and C_3_ is the absorbance of the background group.

### 3.12. Non-Targeted Metabolomics Analysis

Metabolomics analysis was performed by Wekemo Tech Group Co., Ltd. (Shenzhen, China). The 100 μL sample was placed in an EP tube, followed by 400 μL of 80% methanol aqueous solution, vortex shock, an ice bath for 5 min, and then centrifuge at 15,000× *g* and 4 °C for 20 min. A certain amount of supernatant was diluted with mass spectrometry grade water to 53% methanol and then centrifuged at 15,000× *g* for 20 min at 4 °C to collect the supernatant, which was finally injected into LC-MS/MS for analysis. An equal volume of samples was taken from each experimental sample and mixed as QC samples, and 53% methanol aqueous solution was used instead of the experimental samples as blank samples. The pre-treatment process was the same as that of the experimental samples.

Samples were separated by a chromatograph (Vanquish UHPLC) (Thermo Fisher, Munich, Germany) with a C18 column (100 × 2.1 mm, 1.9 μm). The sample was injected using an autosampler with a column temperature of 40 °C and a flow rate of 0.2 mL/min. The mobile phase consisted of eluent A (water containing 5 mM acetic acid and 0.1% formic acid) and eluent B (methanol). The gradient elution program was set as follows: 0–1.5 min, 98% A, 2% B; 1.5–3 min, 15% A, 85%B; 3–10 min, 100% B; 10.1–11 min, 98% A, 2% B; and finally, 11–12 min, 98% A, 2% B. MS/MS was performed on a Q Exactive™ HF (Thermo Fisher, Munich, Germany) using electrospray ionization (ESI) in both positive and negative ion modes. The scanning range was m/z 100–1500; the ESI source settings were as follows: spray voltage: 3.5 kV; sheath gas flow rate: 35 psi; aux gas flow rate: 10 L/min; capillary temp: 320 °C; s-lens RF level: 60; aux gas heater temp: 350 °C; polarity: positive, negative; MS/MS secondary scans are data-dependent scans.

### 3.13. Statistical Analysis

All experiments were performed in triplicate, and data are expressed as mean ± standard deviation. Single-factor ANOVA was performed to determine statistical significance using IBM SPSS Statistics 25. Bar, line, and pie charts were plotted using Origin 2021; box-behnken was designed using Design-Expert 13; and PCA, volcano, and a hierarchical clustering heat map were plotted using the MetaboAnalyst5.0 website (https://new.metaboanalyst.ca/, accessed on 26 September 2023). KEGG enrichment analysis was performed on the Mbrole2.0 website (http://csbg.cnb.csic.es/mbrole2/analysis.php, accessed on 30 September 2023).

## 4. Conclusions

In the present study, the liquid fermentation process of AL using mixed *Bacillus* species was optimized through response surface methodology. Under optimal fermentation conditions, the flavonoid and polyphenol content in the fermentation broth significantly increased, and the free radical scavenging abilities of DPPH and ABTS were effectively enhanced compared to pre-fermentation levels. Non-targeted metabolomics identified 1348 metabolites, of which 829 were classified as differential metabolites. Notably, polyphenols and flavonoids among these differential metabolites played a pivotal role in enhancing the antioxidant activity of FAL. This study confirmed the feasibility of liquid fermentation of AL using complex probiotics from *Bacillus* species, theoretically validated the changes in physicochemical properties and metabolites of AL post-fermentation, and provided new research directions for the further development and utilization of AL.

## Figures and Tables

**Table 1:** The results of the response surface experiment.

Number	A Fermentation Time (h)	B Fermentation Temperature (°C)	C Inoculation Amount (%)	Total Polyphenol Content (mg GAE/g Sample)
1	0	0	0	83.12
2	1	−1	0	80.6
3	0	−1	−1	78.94
4	−1	−1	0	72.98
5	−1	0	−1	80.31
6	1	1	0	74.25
7	−1	0	1	78.69
8	−1	1	0	82.44
9	0	−1	1	81.56
10	0	0	0	83.41
11	1	0	−1	77.92
12	0	0	0	84.06
13	0	1	1	83.67
14	0	0	0	83.12
15	0	0	0	83.55
16	0	1	−1	83.57
17	1	0	1	82.27

**Table 2:** Analysis of variance of the regression model.

Source	Sum of Squares	df	Mean Square	F-Value	*p*-Value	Significance
Model	174.97	9	19.44	51.63	<0.0001	significant
A	0.0481	1	0.0481	0.1276	0.7315	
B	12.13	1	12.13	32.21	0.0008	***
C	3.71	1	3.71	9.86	0.0164	*
AB	62.49	1	62.49	165.95	<0.0001	***
AC	8.91	1	8.91	23.66	0.0018	**
BC	1.59	1	1.59	4.22	0.0791	
A^2^	67.74	1	67.74	179.90	<0.0001	***
B^2^	14.78	1	14.78	39.25	0.0004	***
C^2^	0.5351	1	0.5351	1.42	0.2721	
Residual	2.64	7	0.3765			
Lack of Fit	2.03	3	0.6781	4.51	0.0899	not significant
Pure Error	0.6015	4	0.1504			
Cor Total	177.60	16				

Note: R^2^ = 0.9852; Adj R^2^ = 0.9661; Pred R^2^ = 0.8114; Adeq Precision = 23.6824. *: *p* < 0.05; **: *p* < 0.01; ***: *p* < 0.001.

**Table 3:** Three-factor, three-level design response surface test.

Factors	Level		
	−1	0	1
A. Fermentation time (h)	36	48	60
B. Fermentation temperature (°C)	34	37	40
C. Inoculation amount (%)	1.5	2	2.5

## Data Availability

Data are contained within the article.
